# Ultrastructural Mapping of the Zebrafish Gastrointestinal System as a Basis for Experimental Drug Studies

**DOI:** 10.1155/2016/8758460

**Published:** 2016-06-02

**Authors:** Delfine Cheng, Gerald J. Shami, Marco Morsch, Roger S. Chung, Filip Braet

**Affiliations:** ^1^School of Medical Sciences (Discipline of Anatomy and Histology), The Bosch Institute, The University of Sydney, Sydney, NSW 2006, Australia; ^2^Faculty of Medicine and Health Sciences, Macquarie University, Sydney, NSW 2109, Australia; ^3^Australian Centre for Microscopy & Microanalysis (ACMM), The University of Sydney, Sydney, NSW 2006, Australia; ^4^Charles Perkins Centre, The University of Sydney, Sydney, NSW 2006, Australia

## Abstract

Research in the field of gastroenterology is increasingly focused on the use of alternative nonrodent model organisms to provide new experimental tools to study chronic diseases. The zebrafish is a particularly valuable experimental platform to explore organ and cell structure-function relationships under relevant biological and pathobiological settings. This is due to its optical transparency and its close-to-human genetic makeup. To-date, the structure-function properties of the GIS of the zebrafish are relatively unexplored and limited to histology and fluorescent microscopy. Occasionally those studies include EM of a given subcellular process but lack the required full histological picture. In this work, we employed a novel combined biomolecular imaging approach in order to cross-correlate 3D ultrastructure over different length scales (optical-, X-ray micro-CT, and high-resolution EM). Our correlated imaging studies and subsequent data modelling provide to our knowledge the first detailed 3D picture of the zebrafish larvae GIS. Our results provide unequivocally a limit of confidence for studying various digestive disorders and drug delivery pathways in the zebrafish.

## 1. Introduction

Zebrafish (ZF,* Danio rerio*) exhibit a high degree of resemblance in their genetic profile (69% of the their genes have at least one human ortholog [1]), molecular mechanisms, cell development, and organ physiology to humans [2]. Their small size and translucent nature make them easy to manipulate and observe as a whole animal [3], which contribute to their attractiveness as model organisms for biological experimentation. Accordingly, ZF have been successfully employed to study various physiological and pathophysiological processes in embryogenesis, organogenesis, genomics, and cancerogenesis, as well as research in toxicology and drug studies (for reviews, see [4–6]).

Although ZF have been shown to be a beneficial complementary model to rodent in many research fields, they lack some of the typical mammalian organs such as lung, prostate, skin, and mammary glands. On the other hand, as vertebrates, ZF possess organs and tissues, such as heart, kidney, liver, pancreas, intestinal tract, and brain that display analogous structures and functions to those found in humans [7]. Furthermore, with the advances in genetics, any human tumour type has also been successfully genetically developed in the ZF with similar morphology, gene expression, and signalling pathways [8, 9]. Consequently, despite their infancy in research fields, ZF have already gained distinctive popularity in the fields of genetics and development [10, 11]: they have become a complementary platform to rodent experimental models in preclinical screening studies in the field of translational drug research, in particular, in the assessment of drug compounds delivered to the gastrointestinal system (GIS). These attributes further extended the use of ZF to include the fields of metabolic organ diseases, including cancer [4, 12–14].

The ZF digestive system organogenesis [15–18] and morphogenesis [19–21] have been described by a few, and histological information is available on FishNet [22], the Zebrafish Atlas [23], and The Zebrafish Atlas of Macroscopic and Microscopic Anatomy [24]. However, to-date, the literatures available regarding comparative imaging studies that explore the ZF GIS with different imaging modalities and fine ultrastructural studies of the ZF microarchitecture are particularly scarce, mainly limited to advanced light-laser optical microscopy approaches. Hence, we present here the first full image sets of a 12 dpf ZF larvae from the macro- to the nanometre scales, including detailed information on cellular and subcellular features of the digestive system organs and a comprehensive comparative analysis of ZF GIS, weighted against published literature from rodents.

## 2. Materials and Methods

**Figure 1:**
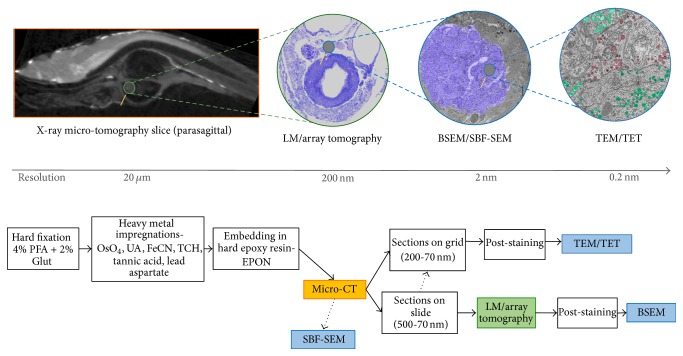
Sample preparation and imaging workflow used for the observation and ultrastructural data correlation of a single zebrafish sample compatible with X-ray micro-CT, LM, and EM imaging modalities. LM imaging modality includes the array tomography technique, whereby serial sections are collected onto a glass slide and imaged using LM and back-scattered EM (BSEM). EM includes TEM, transmission electron tomography (TET), BSEM, and SBF-SEM. This sample preparation protocol not only allows for the sample to be compatible with all the different microscopy platforms but also provides superior ultrastructural preservation of the zebrafish larvae, compared to conventional protocols used for EM.

Firstly, a dedicated sample preparation and imaging workflow was prepared after assessing different experimental approaches, allowing for the subsequent swift imaging of one sample across different microscopy platforms, including X-ray microcomputed tomography (micro-CT), light microscopy (LM), and electron microscopy (EM) (Figure 1, top panel). Secondly, following this workflow, we designed the subsequent sample manipulation processes so the entire sample was retained for whole-mount investigation, allowing for the ability to image multiple areas, multiple times, and across different beam-lines (Figure 1, lower panel).

### 2.1. Zebrafish Animal Model

Zebrafish (wild-type,* Danio rerio*) were maintained at 28°C in a 13 h light and 11 h dark cycle. Embryos were collected by natural spawning and raised at 28.5°C in E3 solution according to standard protocols [25]. Note that, from previous organogenesis studies of the ZF digestive system, it was determined that from 6 days after fertilisation (dpf), when the yolk is exhausted, the digestive functions are comparable to those of an adult fish [15, 19, 21]. Therefore, we studied ZF larvae aged 12 dpf to map the ultrastructure of the GIS. This timeframe is of particular importance as we can take the advantages including the optical translucent properties of the animal and the small size of the fish to facilitate the different sample preparation steps needed, inherent to correlated biomolecular microscopy (i.e., staining and fixation). The sample can be processes as a whole animal (i.e., whole mount), which excludes dissection artefacts and is large enough to easily differentiate the internal organs in LM.

### 2.2. Sample Preparation

For all microscopy examination purposes, ZF larvae at 12 dpf were collected and fixed in 4% paraformaldehyde + 2.5% glutaraldehyde in cacodylate buffer (4% sucrose + 0.15 mM CaCl_2_ in 0.1 M sodium cacodylate buffer), overnight at 4°C. The samples were prepared following a protocol modified from Deerinck et al. [26] and Tapia et al. [27], whereby whole animals were exposed to the following solutions of heavy metal stains and mordanting agents: 2% osmium tetroxide (OsO_4_) + 1.5% potassium ferrocyanide (KFeCN) for 2 h at 4°C, 1% thiocarbohydrazide (TCH) for 20 min, 2% OsO_4_ for 20 min in the dark, 1% aqueous uranyl acetate at 4°C overnight, and Walton's lead aspartate for 30 min at 60°C. The samples were thoroughly washed with distilled water in between staining steps, then dehydrated through a series of ethanol, gradually infiltrated with a series of EPON (hard grade) dilutions over a period of 3 days, and incubated in pure resin over 2 days. Finally, each ZF was carefully positioned, head-down, at the bottom of a BEEM® capsule (bottleneck, size 00), filled with fresh resin, and polymerised at 60°C overnight.

### 2.3. X-Ray Microcomputed Tomography (Micro-CT)

Micro-CT imaging was performed on resin-embedded ZF samples using a Skyscan 1072 system (Bruker microCT, UK), with no filter, operating at 40 kV and over 180 deg rotation angle. The individual X-ray images were reconstructed using NRecon (Bruker microCT, UK) and volumetric data were processed and analysed using Avizo (FEI Software) and IMOD (Boulder, Colorado, USA).

### 2.4. Light Microscopy (LM) and Array Tomography

ZF larvae were orientated sagittal to minimise the block face surface. The head was trimmed off to just behind the eyes and the block was carefully faced up to just behind the inner ears or to the start of the pharyngeal pad, just prior to exposing the oesophagus and the liver. Excess resin around the tissue is trimmed away with a razor blade and a mix of Welwood® glue and xylene (1 : 2) was applied to the sides of the block, following the methods developed by Micheva and Smith [28] and Blumer et al. [29] for array tomography sectioning. We found that applying glue only to the bottom of the trapezoid was sufficient and preferable, as the dried layer of glue from the top and the sides frequently does not cut well and builds up and interferes with the sectioning. This method successfully generated long, uninterrupted serial sections with minimal section loss. A ultramicrotome (Ultracut 7, Leica Microsystems, Heerbrugg, Switzerland) and a histojumbo diamond knife (Diatome, USA) were used to create ribbons of 50 serial sections 0.5 *μ*m thick, which were placed, in order, onto a glass slide previously placed inside the knife boat (Supplementary information 1 in Supplementary Material available online at http://dx.doi.org/10.1155/2016/8758460).

After every ribbon, the slide was dried, stained with 0.5% toluidine blue, and observed with LM for orientation purposes. Once the entire GIS had been sectioned, each section containing the GIS was sequentially imaged using a light microscope (DM6000, Leica, Germany). The ImageJ plugin* StackReg* (Biomedical Imaging Group, EPFL, Lausanne, Switzerland [30]) or the IMOD plugin* midas* (Boulder Lab for 3D Electron Microscopy of Cells, Colorado, USA) was used to either automatically or manually align the images relative to a reference image chosen within the stack (Supplementary information 2). The aligned stack was then used to create 3D models of the GIS organs using 3dMod (IMOD plugin), whereby volumetric and morphometric analysis can be generated. Counting sections from the array tomogram allows for precise localisation (within 0.5 *μ*m) of the different organs, their size, and relative positions with each other.

### 2.5. Back-Scattered Scanning Electron Microscopy (BSEM)

Following LM imaging, the glass slides were carbon-coated and mounted on a stub in preparation for Scanning Electron Microscopy (SEM) imaging. Two lines of silver paint were applied from the top surface of the slides to the stubs to increase conductivity. Sections were then imaged by detection of back-scattered electrons using a SEM (Sigma VP FEG SEM, ZEISS, Germany) operating at 3.8 kV. Consecutive sections were imaged, aligned, and modelled following the same method described previously for array tomography with LM. In this way, dozens or even hundreds of consecutive sections can be imaged over multiple areas, multiple times.

### 2.6. Transmission Electron Microscopy (TEM)

At any time during sectioning for array tomography, ultrathin sections of 70 nm can be collected on 200 mesh copper grids for TEM observation. Typically, after every 50 sections, the ribbons of sections were checked. When an area of interest is apparent on the LM sections, an ultrathin section can be collected at this point which is adjacent to the last section from the last ribbon. Next, retrieved sections on grids were poststained with 2% aqueous uranyl acetate and Reynold's lead citrate solutions for 10 min each and imaged with a TEM (1400 TEM JEOL, Tokyo, Japan), operating at 120 kV.

## 3. Results and Discussion

Currently, ZF have gained exponential momentum as an experimental animal model in biomedical research fields [31–33]. Although the ZF model has shown to be a viable addition to other animal models (e.g., pigs and dogs), and even rodents, they lack some of the typical mammalian organs as outlined earlier. Providentially, ZF possess a GIS that displays an analogous function to humans, including such organs as a liver, pancreas, and gallbladder, with the notable addition of a swim bladder [7]. This unique aspect of the ZF, together with their relative small size and translucent properties—directly benefiting sample processing and imaging—make them very appealing model in the fields of gastroenterology and hepatology. The importance of these features have been convincingly demonstrated throughout the literature, such as for liver development [34] and regeneration [35], hepatocellular carcinoma [36], hepatic nanoparticle-targeting [37], hypertriglyceridemia-mediated pancreatic organ abnormalities [38], drug-induced liver injury [39], intestinal inflammation [40], and gut-associated nutritional programming [41].

Despite the fervent application of ZF in the investigations of various functional aspects of liver and pancreas- and gut-associated diseases, their fine micro- and nanoanatomical structures are relatively unexamined. Further, there is no detailed comparison of ZF GIS to its mammalian and human counterparts. While many teams only apply state-of-the-art live-cell biomolecular optical imaging techniques, limited studies have subsequently verified their findings at the nanoscale, throughout multiple dimensions (i.e., *X*, *Y*, and *Z*). Indeed, high-resolution microscopy is the only way to provide important complementary histological information for many subcellular processes observed using optical microscopy [42–44].

**Figure 2 fig2:**
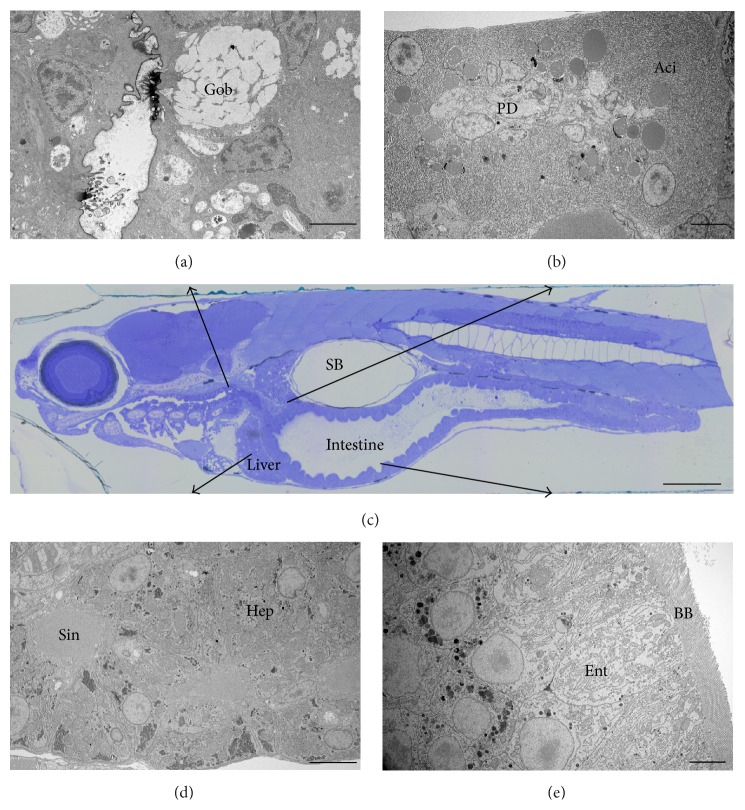
Parasagittal section of a 12 dpf ZF larvae stained with toluidine blue and imaged with light microscopy, showing the different components of the digestive system (c). Corresponding EM images of the different regions include the oesophageal area, rich in goblet cells (Gob) (a), the pancreas, with a pancreatic duct (PD) in the middle and surrounded by acinar cells (Aci) rich in zymogen granules (b). The liver and its hepatocytes (Hep) surrounded by sinusoids (Sin) and its network of bile ducts are shown in (d), as well as the intestine lined with enterocytes (Ent) rich in villi forming the intestinal brush border (BB) in (e). (SB) is the swim bladder. Scale bar = 20 *μ*m (LM) and 5 *μ*m (EM).

**Figure 3 fig3:**
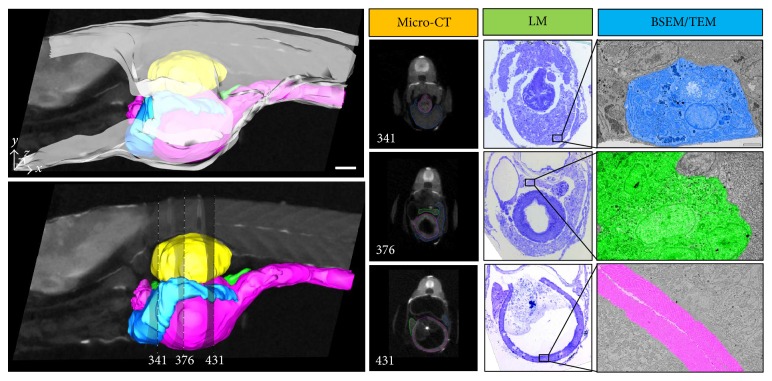
Zebrafish larvae (12 dpf) digestive system imaged using X-ray, LM, and EM (BSEM and TEM). At any positions (here, sections 341, 376, and 431 are shown as examples), micro-CT images and model can be viewed as cross-sections. Corresponding LM images of toluidine blue stained sections (500 nm) can be retrieved by mean of measuring distances from recognisable organs in the X-ray data. Back-scattered SEM images are generated from the same sections as the LM sections. TEM images are generated from adjacent sections from the LM ones. Colour code for micro-CT model: swim bladder (yellow), pancreas (green), intestine (pink), and liver (blue). Colour code for EM images: hepatocyte (blue), islet of Langerhans (green), and intestinal brush border (pink). Scale bars = 100 *μ*m (micro-CT) and 2 *μ*m (TEM).

**Figure 4 fig4:**
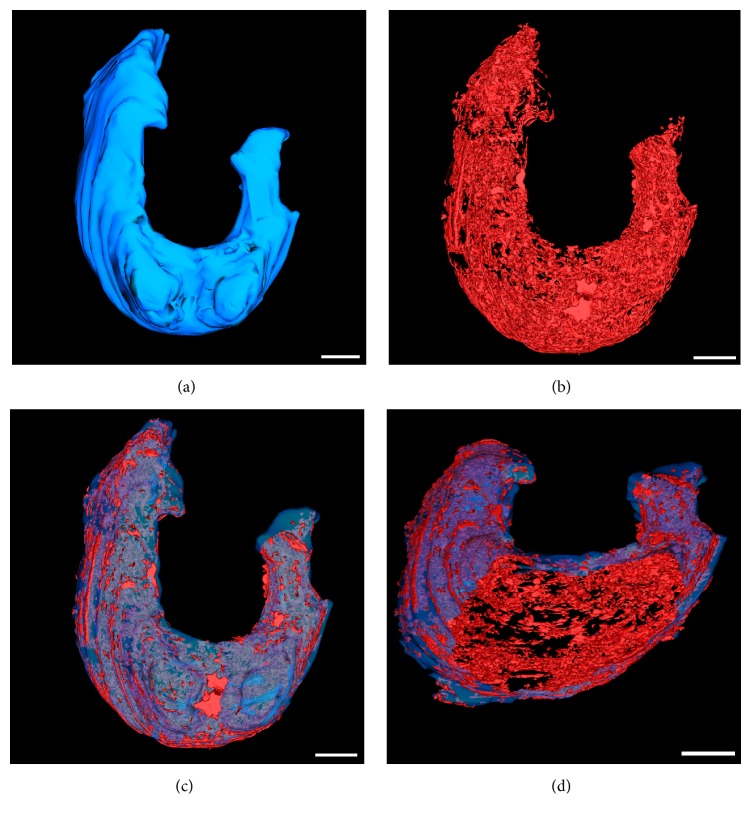
Zebrafish larvae (12 dpf) model of liver (blue) and its vasculature (red), generated by serial LM imaging of 416 consecutive sections of 500 nm. (a) Dorsal view of the liver. (b) Vasculature of the liver (17% of total volume) represented in (a). (c) Combined liver model and its vasculature. (d) Same as (c), viewed from a different angle and clipped opened to visualise the internal vasculature. Liver vasculature was modelled by thresholding the grey values corresponding to the vessels and sinusoids from individual LM images. For full animation, see Supplementary information. Scale bar = 50 *μ*m.

**Figure 5:**
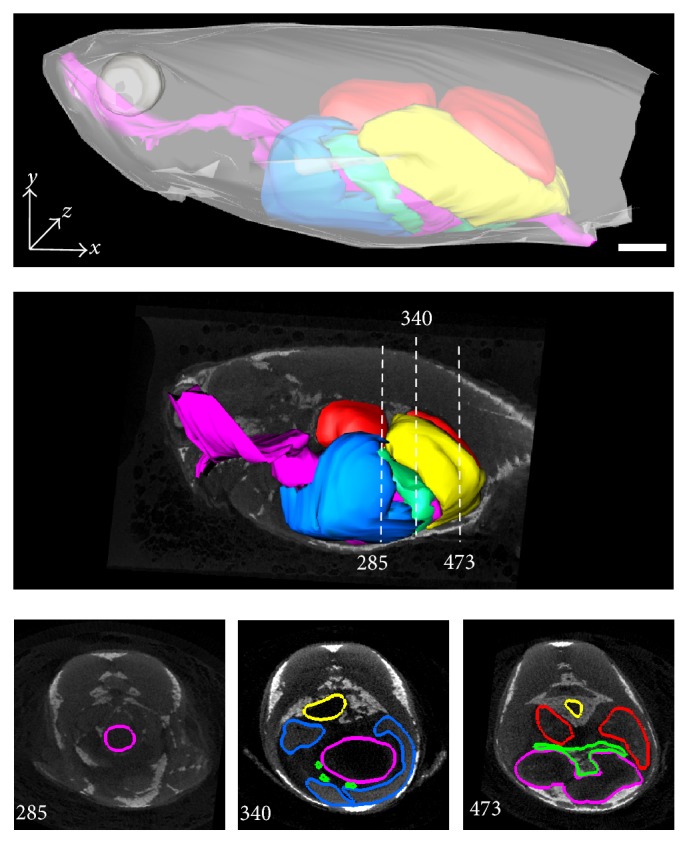
Adult zebrafish digestive system reconstruction by X-ray micro-CT, showing the GIT (pink), liver (blue), pancreas (green), swim bladder (yellow), and oocytes (red). Cross-sections are shown on the bottom line for different positions (here, positions 285, 340, and 473 are used as examples). Scale bar = 20 mm.

In this contribution, we outline an alternative multimodal imaging route to fill in the missing histological pieces of the ZF GIS puzzle (Figure 1). By doing so, we firstly illustrated the unique overall ultrastructural resemblance of the ZF GIS to the mammalian GIS as examined by standard TEM (Figure 2). Next, we expanded those results by employing contemporary three-dimensional (3D) microscopy techniques (Figures 3 [Fig fig4]–5) and finally reviewed our fine structure findings, including our morphometric data, against the existing literature (Table 1).

The sample preparation protocol used in this study was modified from Deerinck et al. [26] and Tapia et al. [27] and was originally developed for the preparation of biological samples for serial-block face sectioning and back-scattered SEM imaging. Although the sample processing time is significantly longer (6-7 days) than that of a conventional sample preparation protocol for EM (3-4 days), we have still opted to base our protocol on the two protocols mentioned previously: the aforementioned series of heavy metals stains and mordanting agents have shown great contrast improvement and conductivity of biological tissues, critical for high-resolution EM imaging, without interfering with conventional LM staining. The use of reduced osmium (RO) with potassium ferrocyanide not only improved membrane preservation but also contrasted glycogen granules in hepatocytes [45, 46]. The addition of sucrose and CaCl_2_ to the buffer further improved the ultrastructural preservation by stabilising the osmolarity [47] throughout the processing. X-ray imaging was used here as a relative quick way to image the GIS in its entirety. Due to the small size of the animal (in average 5 mm × 700 *μ*m), a full scan of the GIS usually takes 2 hours while it would take a few days to scan an entire rat at a comparable resolution. Gross organ sizes and their relative positions within the fish body can be observed. The small sample size and the enhanced contrasting protocol used in this work also made the segmentation and modelling of the internal organs relatively easy. In fact, ZF samples processed with the protocol herein displayed superior contrast relative to samples processed using a standard protocol involving staining with osmium tetroxide only. In fact, previous studies have shown that soft tissue contrast can be enhanced for X-ray micro-CT imaging by heavy metal staining [48, 49]. Imaging of the sample embedded in resin also exhibits a higher signal-to-noise ratio compared to sample immersed in ethanol (Supplementary information 3). This may be related to the enhanced stability of the sample—increased signal—as well as the embedding media—decreased noise—during the scan acquisition. By imaging the entire intact ZF in the micro-CT, information such as 3D connectivity is retained and significant internal tissue damage resulting from sample preparation can be assessed. Since micro-CT is a nondestructive imaging technique, the same sample block can be retrieved and sectioned, and subsequent data can be correlated with histological (i.e., LM) and/or subcellular ultrastructural studies (i.e., EM) (Figure 3), as the sample was processed using a protocol compatible to all the mentioned imaging modalities. Sectioning for array tomography is the most time consuming step: it will take an experienced microtomist 6–8 hours to section through the entire GIS sagittal and collect the ribbons of sections on glass slides, following which the samples are conserved for future LM and EM imaging and analysis.

Micro-CT analysis allowed us to determine that the GIT of a 12 dpf ZF was approximately 2.5 mm long, from the oesophagus to the anus.

The liver, the largest of the digestive glands, plays a pivotal role in the maintenance of metabolic function and excretion [50]. Our micro-CT and array tomography reconstructions showed that the liver is a trilobe organ with a boomerang-like shape and lies ventrally and anterior to the swim bladder, surrounding the intestine (Figure 3). These observations are supported by previous histological observations [20]. In a 12 dpf larvae, we measured the liver to be approximately 25 *μ*m (anterior-posterior diameter) and occupying an average volume of 5.10^6^ *μ*m^3^ (approximately 2% of a 12 dpf ZF total body volume) within which 17% is occupied by blood vessels (Figure 4). Compared to a rat liver, a ZF liver volume is hence calculated to be 2 400 000 times smaller in volume and 2.2% less vascularised. From previous histological observations, the ZF liver is similar to that of other teleosts [51]: there are no portal triads nor hepatocytes arranged in plates but rather tubules of hepatocytes among portal veins, hepatic arteries, large biliary ducts, and sinusoids, which are distributed stochastically within the parenchyma [52]. Morphologically, the hepatocytes we analysed were polygonal, measured 17 *μ*m in diameter (Figure 3), and formed tubules of small bile ducts, derived from the bile canaliculi, which was intrahepatic and had an average diameter of 2.2 *μ*m. EM data showed vessels and sinusoids also lined with a monolayer of endothelial cells, exhibiting fenestrations varying from 80 to 230 nm in diameter, which means they can be up to double the size of the liver endothelial cells fenestrations reported in rats [53]. Notable was the apparent absence of Kupffer cells throughout the hepatic sinusoids, a distinct point of difference to mammalian livers.

Like in other animals who possess a pancreas, the ZF pancreas is a dichotomic organ and the site of glucohomeostasis [21, 54]. In the ZF, the pancreas is diffused and located around the liver and intestine: the exocrine part is mainly composed of pancreatic ducts and acinar cells. From our correlative imaging studies, the acinar cells were polyhedral, measured 8–10 *μ*m in diameter, and were populated with relatively large zymogen granules (2–2.5 *μ*m, compared to 500–800 nm found in rats) which discharge their contents into the pancreatic ducts to form the pancreatic juice. A principal islet of Langerhans (or Brockman body), identified in the pancreas head and measuring 50 *μ*m in diameter, along with scattered secondary islets forms the endocrine component. The islets composition is as found in mammals, whereby a rich network of blood vessels surrounds neuroendocrine cells, namely, alpha-cells, beta cells, and delta-cells, which, respectively, produce the hormones glucagon, insulin, and somatostatin. Gamma and polypeptide producing- (PP-) cells appear to be absent (Figure 3) [24]. From our light microscopy analysis, the vasculature within the principal islet occupied approximately 12% of the islet. Glucagon and insulin granules were smaller than those found in the rat, measuring, respectively, 182 nm and 160 nm compared to 172 nm and 207 nm.

The gastrointestinal tract (GIT) is a long 3-fold tube, starting from the oesophagus to the anus, with morphological similarities to mammals: a simple columnar epithelium with folds (no real villi) including enterocytes, goblet cells, and endocrine cells. On the apical side, microvilli are present and the enterocytes are joined by tight junctions, which we determined by TEM to be 200–400 nm across. In the ZF, the oesophagus is short and muscular and is mainly composed of goblet cells and taste buds as it is the first site of enzymatic digestion. The intestine follows the oesophagus and histologists have divided it into 3 parts: the intestinal bulb, the midintestine, and the posterior intestine which can be differentiated by their shape, density of goblet cells, and the length of microvilli forming the brush border [18, 55]. Paneth cells, crypts, and organised lymphoid structures are absent across the entire intestine [55]. The intestinal bulb plays the role of food storage and fat absorption [24]. From our X-ray and LM observations, the “bulb” was dilated up to 80 *μ*m wide; however, it could probably extend more following a large meal. Enterocytes were the main cell types and our analysis revealed that the enterocytes at the very beginning of the intestine have the longest microvilli (up to 7.5 *μ*m long) after which they shorten to about 2-3 *μ*m within the intestinal bulb (Figure 3). The midintestine is narrow and believed to be the site of protein absorption [19, 56]. Enterocytes in this area presented microvilli of similar length to those found in the intestinal bulb and were surrounded by more goblet cells than in the intestinal bulb. In the posterior intestine, goblet cells were still present but enterocytes presented short or few microvilli. In fact, most of the digestion process has already taken place and this part is mainly involved in osmoregulation [18].

Finally, we demonstrated that our whole-mount multimodal imaging approach could also be applicable to adult ZF (Figure 5). The versatile imaging workflow presented in this study validates the anatomical fine structures of the adult ZF GIS and compares the larvae ZF GIS systematically to the rodent experimental models (Table 1).

## 4. Conclusion

**Figure 6:**
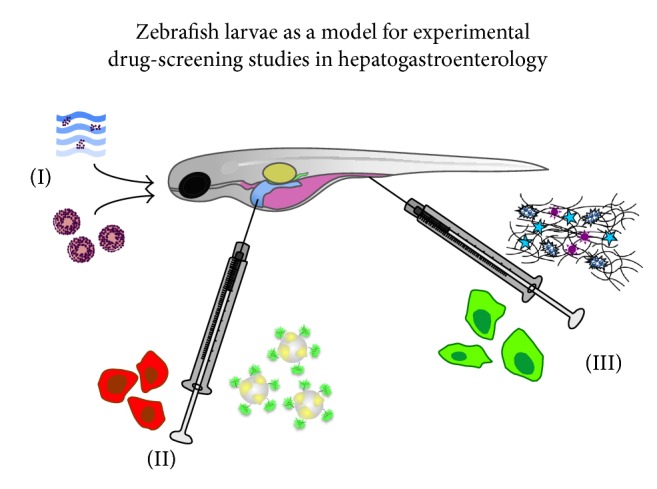
Illustration showing the different routes of administration possible in zebrafish larvae to study the uptake, transport, metabolism, and efficacy of therapeutic drug- and/or cell-based approaches. (I) Indirect administration of complexes dissolved in water or administered directly via the oral route, mixed with food pellets. (II-III) Local and targeted microinjection of fluorescent macromolecular complexes at the site of interest or the use of microcapillary needles to deposit genetically modified cells within the digestive glands (II) or intestines (III). Note that those three administering routes are typically employed in routine preclinical screening studies in rodent models and human studies as well underpinning the relevance of the zebrafish model to investigate the pharmacology, toxicology, and effectiveness of new therapeutic interventions. Taking advantage of the optical translucent properties of the larvae, subsequent whole-mount live-cell imaging allows systematic monitoring of the treatment regimes using fluorescent navigation. The results can be combined with correlated electron microscopy techniques as depicted under Figures 1 [Fig fig2] [Fig fig3]–4. Colour legend for the zebrafish: swim bladder (yellow); stomach and intestines (purple); liver (blue); pancreas (green).

The sample preparation protocol presented here offered not only great versatility—compatible with X-ray, light, and electron microscopy—but also optimum ultrastructural preservation of ZF larvae as well as improved sample contrast and conductivity, required for high-resolution X-ray and EM imaging. The workflow can be easily adapted to incorporate* in vivo* fluorescence or fluorescently labelled structures by adding a fluorescent live imaging step prior to sample processing. The concept of Correlative Light and Electron Microscopy (CLEM) can then be applied to complement dynamic functional information with high-resolution ultrastructural details, on the same sample. The use of tracers or fiducial markers such as laser etching techniques [57] or carbon-coating of pattern on glass slides [58, 59] can facilitate the relocalisation of the area, cell,or subcellular structure of interest between the macro- and nanometre scales imaging. This workflow presents a real advantage in the fields of research aiming at exploring drug transport and xenobiotic metabolism within the digestive glands (e.g., liver and pancreas) or studying and modifying malignant cell behaviour via novel anticancer therapeutic approaches (e.g., colon, pancreas, and liver cancer) (Figure 6). In fact, the low cost, small size, and relative speed at which drugs can be tested in ZF have already made it a popular model for the aforementioned studies. By following the entire workflow, gross differences between experimental and control fish can be rapidly determined by X-ray micro-CT, while detailed analysis of the drug treatment upon pathology can be subsequently evaluated, on the same sample. While all imaging modalities might not be available within a certain research laboratory or institute, this workflow can be adapted to only incorporate the imaging instrumentation available. Since the sample has been prepared to accommodate all imaging techniques aforementioned, one can skip imaging modalities which are unavailable without affecting the next. Also, depending on the study, one can also limit the imaging techniques to only include the ones needed to answer the biological question.

Our comparative microscopy maps and concomitant delivery of 3D imaging models of the ZF digestive system provide a comprehensive overview of the ZF GIS and establish the much needed foundation as well as a limit of confidence in the use of ZF for future research in gastrodigestive related illnesses.

## Supplementary Material

Supplementary Material include technical notes related to array tomography serial sections production and automated alignment using the ImageJ plugin StackReg. A comparative review of different sample preparation protocols and their effect on X-ray micro-CT imaging contrast are also presented. Finally, a 3D animation of a 12 dpf zebrafish liver and its vasculature as determined by thresholding methods from serial light microscopy sections.

## Figures and Tables

**(a):** 

Zebrafish liver		Zebrafish pancreas		Zebrafish gut	
Size	*12 dpf*: volume = 5 061 840 *µ*m^3^. Transverse diameter = 349 *µ*m. Anterior-posterior diameter = 520 *µ*m *Adult*: volume = 0.535 mm^3^. Transverse diameter = 2.08 mm. Anterior-posterior diameter = 1.64 mm. (In males, ∼2.10% of total body weight. In females, ∼4.51% of total body weight)	Size	*12 dpf*: Volume = 1 738 670 *µ*m^3^. Transverse diameter = 290 *µ*m. Anterior-posterior diameter = 500 *µ*m *Adult*: volume = 0.3 mm^3^. Transverse diameter = 1.9 mm. Anterior-posterior diameter = 2.5 mm	Size	Long tube of about 400 *µ*m. The interior is also folded and lined with a layer of simple columnar epithelium

Location	Posterior to inner ears and pharyngeal pad, anterior to intestinal bulb	Location	Between the intestine and the swim bladder. The head starts right posterior to the liver	Location	From esophagus to anus

Shape	3 lobes, boomerang shape. The left lobe, the largest, crosses the midline, under the swim bladder and the right lobe extends ventrally towards the head of the pancreas	Organisation	The pancreas is very diffused and acinar cells are scattered	*Oesophagus*	Located under the trachea. Short and muscular

Organisation of hepatocytes	Tubular	*Endocrine tissue*	One principal islet (or Brockman body) in the head (50 *μ*m diameter). 2-3 secondary islets surround the principal islet	Composition	Mostly goblet cells and a few enterocytes

Composition	Hepatocytes, endothelial cells, bile duct epithelial cells. Kupffer cells seem to be absent	Beta cells	Insulin granules (160 nm)	Role	First place of enzymatic digestion

*Hepatocytes*	65% of total liver volume	Alpha cells	Glucagon granules (182 nm)	*Intestinal bulb*	Dilated, up to 80 *µ*m in diameter

Size	Polygonal, 14–17 *µ*m in size. Hepatocytes form plates, lined with sinusoids and biliary ducts	Other-cells	Delta-cells produce somatostatin	Composition	Enterocytes with very long microvilli at the beginning (up to 7.5 *µ*m long and 115 nm in diameter) and then shortened to 2-3 *µ*m long

Organelles	Nucleus, rER, mitochondria, Golgi apparatus, glycogen, lipid droplets, lysosomes	*Vascular system*	Rich vasculature (12% of the islet)	Role	Fat absorption

*Biliary system*	18% of liver volume	*Exocrine tissue*	Production of pancreatic digestive enzymes	*Midintestine*	Narrow, folded 3 times on itself and measures 120–140 *µ*m in diameter

Bile canaliculi	Presence of microvilli on the surface. Diameter = 2.22 *µ*m	*Acinar cell*	Polyhedral, 8–10 *µ*m in size, surrounding a central acinar duct	Composition	More goblet cells than in the intestinal bulb. Enterocytes have shorter microvilli (2-3 *µ*m long, 115 nm in diameter)

Bile ducts	Intrahepatic, and extrahepatic ducts	Organelles	Zymogen granules (2.30 *µ*m), nucleus, ER, mitochondria	Role	Proteins absorption

*Vascular system*	17% of liver volume	*Pancreatic system*	Main pancreatic ducts through the middle of the pancreas head	*Posterior intestine*	Narrow and measures 80–90 *µ*m in diameter

Endothelial cells	Fenestrated, average diameter = 130 nm.			Composition	Rare or no microvilli
				Role	Osmoregulation

**(b):** 

Rat liver		Rat pancreas		Rat gut	
Size	Transverse diameter = 7.5–8 cm, superior-inferior diameter = 2.8–4.2 cm, anterior-posterior diameter = 2.2–2.5 cm. 5% of total body weight, mean weight = 13.6 g	Size	Weight = 804 mg	Size	The long tube measures up to 2 m in length

Location	Upper right portion of the abdomen, beneath the diaphragm and above the stomach. A small portion extends into the upper left quadrant	Location	In the cranial abdominal cavity, between the stomach and the small intestine	Location	From mouth to anus

Shape	4 lobes: left, middle, right, and caudate	Organisation	Diffused, lobulated in appearance and divided into 3 parts: biliary, duodenal, and gastrosplenic	*Oesophagus*	Long tube from mouth to stomach, approx. 20 cm long

Organisation of hepatocytes	Lobular	*Endocrine tissue*	2–3.5% of the pancreas. 5000 islets of Langerhans scattered within the organ	Composition	Stratified squamous epithelium

Composition	Hepatocytes, endothelial cells, bile duct epithelial cells, Kupffer cells	Beta cells	65–80% of the islet. Production of insulin granules (207 nm)	Role	Transport food from mouth to stomach

*Hepatocytes*	80.6% of total liver volume	Alpha cells	15–20% of the islet. Production of glucagon granules (172 nm)	*Stomach *	*Functions are comparable to the ZF intestinal bulb*, approx. 6 cm long

Size	Polyhedral, 27 *µ*m in diameter. Overlapping plate-like sheets (trabeculae) form the three-dimensional structure of the liver lobule	Other-cells	Delta-cells (3–10% of the islet) produce somatostatin. PP-cells (3–5% of the islet) produce pancreatic polypeptides	Composition	Enterocytes are the main cell types (90% of the villi surface)

Organelles	Nucleus (25% are binucleate), mitochondria, rER, sER, lysosomes, Golgi apparatus, peroxisomes, lipid droplets, free ribosomes, lipoproteins, glycogen, polyosomes	*Vascular system*	Rich vasculature (10% of the islet)	Role	Storage of food, start of enzymatic digestion

*Biliary system*	0.2% of liver	*Exocrine tissue*	95% of the pancreas	*Small intestine*	*Functions are comparable to the midintestine*, approx. 1–1.5 m long

Bile canaliculi	Presence of microvilli on the surface. Diameter = 1.5 *µ*m	*Acinar cell*	Polyhedral, 10 *µ*m in diameter, surrounding a central acinar duct.	Composition	Divided into 3 parts: duodenum, jejunum, and ileum. Enterocytes are the main cell types

Bile ducts	Intrahepatic, interlobular, and extrahepatic bile ducts	Organelles	Zymogen granules (500–800 nm), nucleus, ER, mitochondria	Role	Absorption of nutrients

*Vascular system*	19.2% of liver volume	*Pancreatic system*	Anterior pancreatic duct (main duct) occupies 50–60% of the pancreas. Draining the pancreatic juice to the stomach and small intestine	*Large intestine*	*Functions are comparable to the posterior intestine*, approx. 22–26 cm long

Endothelial cells	Fenestrated, average diameter = 6.5 *µ*m			Composition	Enterocytes
				Role	Absorption of water and left over digested nutrients
