# Supplementary material for: Ultrastructural Mapping of the Zebrafish Gastrointestinal System as a Basis for Experimental Drug Studies
Source: Biomed Res Int. 2016 Jun 2;2016:8758460. doi: 10.1155/2016/8758460 (PMC4909927; doi:10.1155/2016/8758460)
Supplement: Supplementary file 1 — Supplementary Material include technical notes related to array tomography serial sections production and automated alignment using the ImageJ plugin StackReg. A comparative review of different sample preparation protocols and their effect on X-ray micro-CT imaging contrast are also presented. Finally, a 3D animation of a 12 dpf zebrafish liver and its vasculature as determined by thresholding methods from serial light microscopy sections. [file 8758460.f1.pdf]

SUPPLEMENTARY INFORMATION 1

In case the glass slide used for collecting sections for array tomography was too hydrophilic, water would occasionally run in between the knife and the slide, slowly lowering the water level in the boat during the sectioning. To prevent water from escaping the knife boat by capillarity, a thin strip of nail polish was proactively painted and let to dry across the width of the glass slide, at the level where the water touches the knife boat.

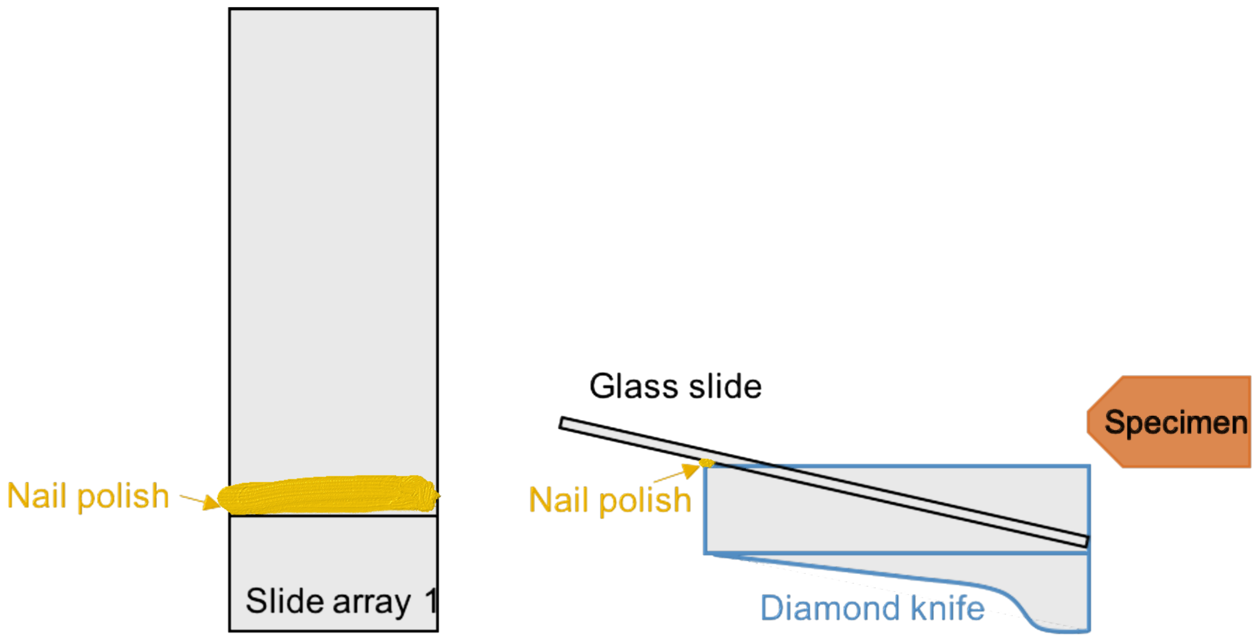

Schematic for the application of nail polish to the glass slide used to collect ribbons of serial sections. *The nail polish is applied at the interference between the glass slide and the knife boat and prevents the water from escaping the knife boat, by capillarity.*

SUPPLEMENTARY INFORMATION 2

StackReg is an easy way to automatically align the images within a stack. However, it has limitations in terms of amount of misalignment present within the image stack. When the amount of misalignment within the stack is significant, Midas is used instead for manual alignment of individual images, one-by-one. To save time, one can roughly align the stack using *Midas*, create a new stack then run the roughly aligned stack with StackReg [30].

SUPPLEMENTARY INFORMATION 3

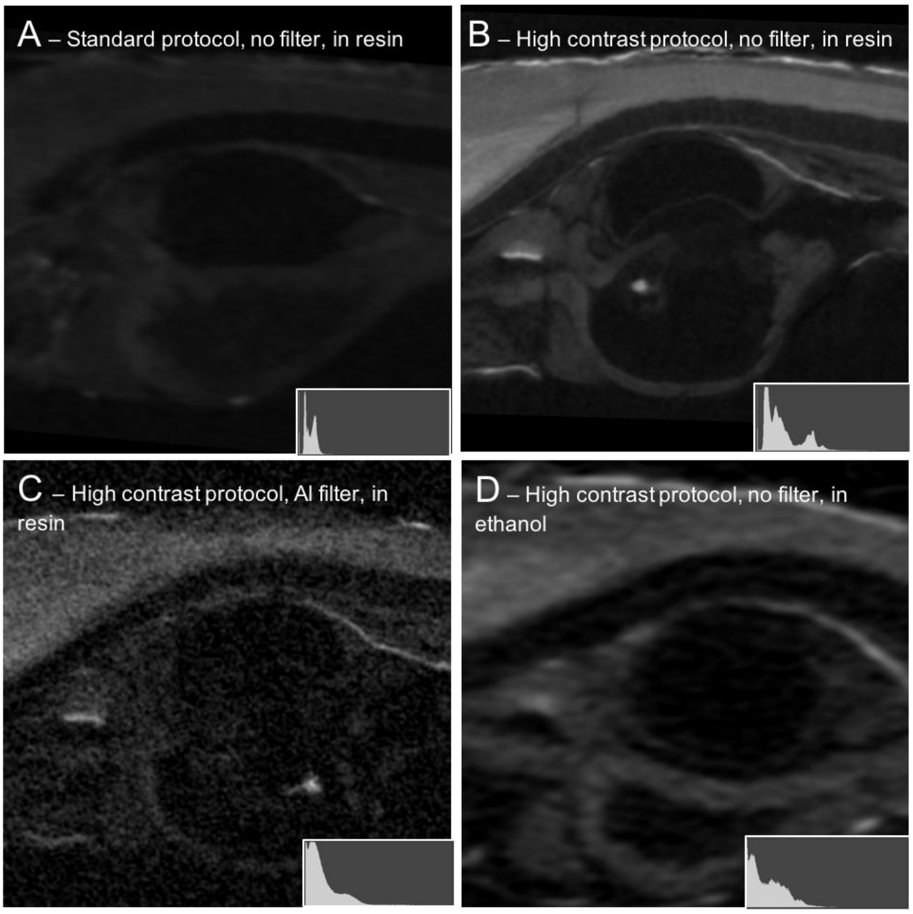

Comparison of sample processing protocol (standard and high contrast protocols), embedding mediums (EPON resin and ethanol) and micro-CT imaging conditions (no filter and 1.00 mm Aluminium filter). *To standardise the comparison, all images were thresholded to HU values 0 - 0.84 and each images is accompanied by its histogram showing the distribution of grey values. HU values 0 – 0.84 was the widest range of signal we obtained from the 4 conditions and was obtained from imaging zebrafish samples processed following the high contrast protocol described in this work, embedded in EPON resin and without any metal filter (B). In fact, it rendered the best signal-to-noise ratio and contrast when compared to zebrafish processed using standard EM protocol (1% Osmium tetroxide as the only heavy metal staining agent) (A), when imaged through a 1.00 mm Aluminium filter (C) or when imaged in ethanol (D). (A), (C) and (D) all presented either less signal-to-noise ratio (C) or less contrast (A) compared to (B).*

SUPPLEMENTARY INFORMATION 4

3D animation of a 12 dpf ZF liver and its vasculature, reconstructed using array tomography technique and light microscopy, as well as thresholding of the grey values corresponding to the blood vessels and sinusoids in the liver.
